# Bleeding and Recurrence in Patients with Venous Thromboembolism with Changing Anticoagulant Treatment Patterns: Findings from the TROLL Registry

**DOI:** 10.1055/a-2832-8297

**Published:** 2026-03-24

**Authors:** Katarina Glise Sandblad, Mazdak Tavoly, Waleed Ghanima, Camilla T. Jørgensen

**Affiliations:** 1 Department of Molecular and Clinical Medicine, Institute of Medicine, Sahlgrenska Academy, University of Gothenburg, Gothenburg, Sweden; 2 Department of Medicine, Geriatrics and Emergency Medicine, Sahlgrenska University Hospital/Östra, Region Västra Götaland, Gothenburg, Sweden; 3 Department of Research, Østfold Hospital, Sarpsborg, Norway; 4 Department of Acute Medicine and Geriatrics, Sahlgrenska University Hospital, Gothenburg, Sweden; 5 Institute of Clinical Medicine, University of Oslo, Østfold, Norway; 6 Department of Emergency Medicine, Østfold Hospital, Sarpsborg, Norway

**Keywords:** venous thrombosis, pulmonary embolism, deep vein thrombosis

## Abstract

**Background:**

Treatment for venous thromboembolism (VTE) has shifted from vitamin K antagonists (VKAs) to direct oral anticoagulants (DOACs), with increased use of indefinite therapy. However, data on bleeding and recurrence outcomes associated with these changes remain limited.

**Objective:**

To evaluate the risk of major bleeding (MB), clinically relevant nonmajor bleeding (CRNMB), and recurrence with changing treatment patterns.

**Methods:**

Cancer-free VTE patients were identified in the Venous Thrombosis Registry in Østfold Hospital, Norway, from January 2005 to May 2020, and categorized into period 1 (2005–2012), period 2 (2013–2016), and period 3 (2017–2020). MB and CRNMB rates during anticoagulant treatment, and recurrence rates after anticoagulant discontinuation were assessed. Subhazard ratios (sHRs) were calculated using Fine-Gray analysis for time-period comparisons.

**Results:**

Among 3,839 patients (48% female, median age: 67 years; IQR, 54–78), VKA use decreased from 76% in period 1 to 2% in period 3, while DOAC use increased from 1 to 85%. The MB rate ranged from 5.6 (95% confidence interval [CI]: 3.9–6.4) per 100 person-years in period 2 to 3.1 (2.3–4.2) in period 3. The CRNMB rate increased from 5.4 (95% CI: 4.3–6.9) per 100 person-years in period 1 to 7.7 (6.3–9.5) in period 3, sHR: 1.87 (1.36–2.56). Conversely, the recurrence rate decreased from 5.1 (95% CI: 4.6–5.6) per 100 person-years after anticoagulant discontinuation in period 1 to 3.9 (3.2–4.9) in period 3, sHR: 0.51 (0.41–0.65).

**Conclusion:**

VTE recurrences after anticoagulant discontinuation have declined, likely due to indefinite treatment in patients at high risk of recurrence, while CRNMB rates have increased over time.

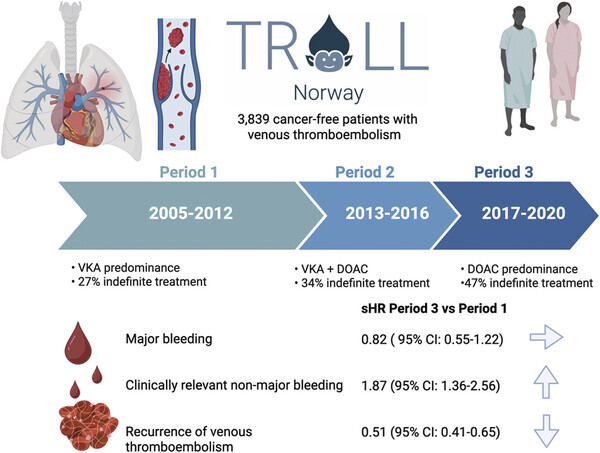

## Introduction

Direct oral anticoagulants (DOACs) are recommended as standard treatment for venous thromboembolism (VTE), 1 2 and over the last decade, a gradual shift from vitamin K-antagonist (VKA) use to DOACs has occurred. 3 Concomitantly, guideline recommendations concerning treatment duration have been updated. The lower risk of bleeding has changed the risk-benefit balance, and indefinite or extended treatment is recommended for a larger number of patients. 1 4 5 While the decline in fatal VTE during the 1-year period following a first-time pulmonary embolism (PE) suggests that this change may have had a favorable effect, 6 the overall impact of treatment changes on bleeding and VTE recurrence over time is scarcely studied. Previous studies on this topic faced methodological limitations, such as combining data from cancer and noncancer patients and failing to evaluate clinically relevant nonmajor bleeding (CRNMB), 7 or relying on imprecise bleeding assessments. 8 Additionally, we found no prior study including a European patient population.

While large-scale national register studies are suitable for evaluating real-world VTE outcomes such as hospitalization for bleeding or mortality, they are not ideal for assessing outcomes such as less severe bleedings or recurrences. 9 10 11 Cohort studies with validated recurrent and bleeding events, such as the Venous Thrombosis Registry in Østfold Hospital (TROLL), a single-center registry, 12 are more appropriate for assessing these outcomes.

We aimed to compare event rates and 6-month and 2-year incidence of major bleeding (MB) and CRNMB in cancer-free patients diagnosed with VTE during anticoagulant treatment in the period 2005 to 2012 with those diagnosed in 2013 to 2016 and 2017 to 2020. The secondary aim was to compare the risk of recurrence after discontinuation of anticoagulant treatment. We hypothesized that the bleeding risk during anticoagulant treatment and recurrence risk after treatment discontinuation decreased over time as treatment shifted from VKAs to DOACs and the use of indefinite treatment increased.

## Materials and Methods

### Study Setting and Population

The TROLL, Norway, includes patients with objectively confirmed incidental or symptomatic venous thrombosis at Østfold Hospital. Detailed information on the registry has been reported previously. 12 Briefly, patients are identified in the hospital ward or at the thrombosis outpatient clinic and asked to participate in the study. Written informed consent is obtained from all patients. Hospital records are searched at regular intervals by dedicated study personnel to identify patients with confirmed VTE who have died during hospitalization or who, for any reason, are unable to come to the outpatient clinic. For these patients, informed consent is obtained via post, except for deceased patients. Most patients are followed in the outpatient clinic throughout the anticoagulant treatment period, and the treating physician or nurse records baseline characteristics and outcomes such as bleeding. For recurrent events after treatment discontinuation, patients are recorded and followed up as for initial events. Between 10 and 20% of patients are unable to attend follow-up. Data for these patients are collected from medical chart review performed by dedicated study personnel to identify and register bleedings and recurrences.

We included all patients with VTE (defined as PE, lower extremity deep vein thrombosis [DVT], or upper-extremity DVT [UEDVT]) without known cancer (newly diagnosed, metastatic, inoperable, or treated with chemotherapy, radiotherapy, and/or surgery within 6 months prior to or 3 months after the VTE) who were registered in TROLL between January 2005 and May 2020. Patients were categorized into three time periods: period 1 (2005–2012), period 2 (2013–2016), and period 3 (2017–2020), corresponding to the time period during which they were included in the registry. The included baseline variables comprised sex, age, body mass index calculated in kg/m ^2^ (BMI), surgery, trauma, immobilization, oral contraceptives, hormone replacement therapy, pregnancy or puerperium, long-haul flights, previous VTE, known thrombophilia, and unprovoked VTE.

Anticoagulant treatment was categorized into three categories: DOACs (rivaroxaban, apixaban, dabigatran, and edoxaban), VKA, and low-molecular-weight heparin (LMWH). The duration of treatment was grouped into 0 to 6 months, 6 to 12 months, over 1 year (but not indefinite), and indefinite treatment.

### Outcomes

MB and CRNMB were defined according to the criteria established by the Control of Anticoagulation Subcommittee of the International Society on Thrombosis and Haemostasis (ISTH). 13 14 Recurrence was defined as a new VTE (in a new vein or extension from the initial VTE) during follow-up, objectively confirmed by imaging (computed tomography pulmonary angiography, compression ultrasonography, venography, ventilation/perfusion scintigraphy, magnetic resonance imaging), or autopsy. All bleeding and recurrence outcomes were confirmed by manual chart review by dedicated study personnel. Follow-up ended in December 2023.

### Statistical Analysis

Categorical variables were described by numbers and percentages, while medians and interquartile ranges were used to describe continuous variables. The bleeding and recurrence risks were expressed as event rates per 100 patient-years with 95% confidence intervals (CI), calculated by dividing the number of events by the total number of patient-years at risk. Cumulative incidences of bleeding and recurrence were estimated using the Fine-Gray subdistribution hazard model, accounting for the competing risk of death at 6 months, 1 year, and 2 years. The subhazard ratios (sHR) compared with period 1 for bleeding and recurrence were reported. The Fine-Gray subdistribution hazard model was chosen over the Cox proportional hazards model to account for death as a competing risk. 15 Differences in bleeding and recurrence rates across the different time periods were assessed using the Wald chi-square test. Curves for the cumulative incidence of bleeding and recurrence were generated using the cumulative incidence function. For MB events, follow-up time was defined as the period from the date of VTE diagnosis until the occurrence of MB, migration out of the hospital's catchment area, death, discontinuation of anticoagulant treatment, or December 1, 2023, whichever occurred first. The same approach was applied for CRNMB events, with analyses censored for both MB and CRNMB. For VTE recurrence, follow-up time was calculated from the date of discontinuation of anticoagulant treatment until recurrence, migration, death, or end of follow-up (December 1, 2023), whichever occurred first. All statistical analyses were performed using Stata Statistical Software, Version 18.0 (StataCorp LLC, College Station, Texas, United States).

This study was approved by the Regional Committee for Medical and Health Research Ethics (Reference Number 267223), which allowed the use of data from individuals who had signed an informed written consent or were deceased.

## Results

By May 2020, a total of 4,890 patients were eligible for research in the TROLL registry. A total of 1,051 participants (21.5%) were excluded for the following reasons: abdominal vein thrombosis (110 cases), cerebral sinus vein thrombosis (10 cases), other venous thrombosis (20 cases), and active cancer (911 cases). Thus, 3,839 patients were included, with a median age of 67 years (IQR, 54–78), and 1,845 (48.1%) were female ( Table 1 ). Among these patients, 2,008 (52.3%) had PE, 1,737 (45.3%) had DVT, and 94 (2.4%) had UEDVT.

**Table 1:** Baseline characteristics for all patients and patients grouped by time period, as well as anticoagulant treatment choice and treatment duration

	All patients	2005–2012	2013–2016	2017–2020
Total number	3,839	*n* = 1,523	*n* = 1,167	*n* =1,149
Female, *n* (%)	1,845 (48.1)	719 (47.2)	588 (50.4)	538 (46.8)
Age, median (IQR)	67 (54–78)	65 (51–77)	69 (56–80)	66 (54–76)
BMI, median (IQR)	26.9 (24.2–30.5)	27.0 (24.3–30.1)	26.8 (24.0–30.5)	26.9 (24.0–30.8)
Surgery, *n* (%)	649 (16.9)	251 (16.4)	185 (15.9)	213 (18.5)
Trauma, *n* (%)	393 (10.2)	165 (10.8)	119 (10.2)	109 (9.5)
Oral contraceptives, *n* (%)	102 (2.7)	44 (2.9)	25 (2.1)	33 (2.9)
Hormone replacement therapy, *n* (%)	86 (2.2)	30 (2.0)	26 (2.2)	30 (2.6)
Pregnancy or puerperium, *n* (%)	54 (1.4)	31 (2.0)	13 (1.1)	10 (0.9)
Long-haul flights, *n* (%)	306 (8.0)	125 (8.2)	81 (6.9)	100 (8.7)
Previous VTE, *n* (%)	386 (10.1)	207 (13.6)	97 (8.3)	82 (7.1)
Known thrombophilia, *n* (%)	362 (9.4)	171 (11.2)	93 (8.0)	98 (8.5)
VTE in first-degree relatives, *n* (%)	504 (13.1)	180 (11.8)	128 (11.0)	196 (17.1)
Unprovoked, *n* (%)	2,374 (61.8)	896 (58.8)	748 (64.1)	730 (63.5)
VTE location				
PE	2,008 (52.3)	728 (47.8)	647 (55.4)	633 (55.1)
DVT	1,737 (45.3)	762 (50.0)	489 (41.9)	486 (42.3)
UEDVT	94 (2.4)	33 (2.1)	31 (2.7)	30 (2.6)
Patients with anticoagulant treatment				
Total number	3,812 a	1,510	1,154	1,148
LMWH, *n* (%)	790 (21)	340 (23)	308 (27)	142 (12)
VKA, *n* (%)	1,418 (37)	1,154 (76)	239 (21)	25 (2)
DOAC, *n* (%)	1,604 (42)	16 (1)	607 (53)	981 (85)
Rivaroxaban, *n* (%)	1,142 (30)	16 (1)	531 (46)	595 (52)
Apixaban, *n* (%)	451 (12)	0	73 (6)	378 (33)
Edoxaban, *n* (%)	4 (0)	0	0	4 (0)
Dabigatran, *n* (%)	7 (0)	0	3 (0)	4 (0)
Treatment duration				
0–6 mo	1,093 (29)	393 (26)	340 (29)	360 (31)
6–12 mo	1,037 (27)	542 (36)	324 (28)	171 (15)
Over 1 y, not indefinite	338 (9)	169 (11)	96 (8)	73 (6)
Indefinite	1,344 (35)	406 (27)	394 (34)	544 (47)

Abbreviations: DVT, deep vein thrombosis; PE, pulmonary embolism; UEDVT, upper extremity deep vein thrombosis; VTE, venous thromboembolism.

a No treatment: 15, Thrombolysis or unfractionated heparin only: 10, unknown: 2.

**Fig. 1:**
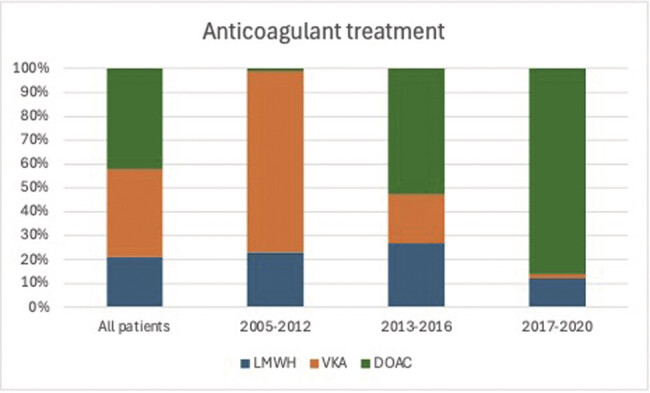
The proportion of VTE patients started on treatment with direct oral anticoagulants (DOACs), vitamin K-antagonists (VKAs), and low-molecular-weight heparins (LMWH) in each time period.

DOAC usage increased from 1% in period 1 to 85% in period 3, while VKA usage decreased from 76 to 2% ( Fig. 1 ; Table 1 ). An increasing percentage of patients received 0 to 6 months of treatment (from 26% in period 1 to 31% in period 3) or indefinite treatment (27–47%), while the 6 to 12 months treatment duration became less common, decreasing from 36 to 15%. Overall, 27 patients did not receive any of the registered anticoagulant treatments. Of these, 15 received no treatment due to high bleeding risk, died before treatment, or had VTE diagnosed at autopsy. Ten patients received thrombolysis or unfractionated heparin but died before another treatment could be started. Two patients received blinded study drugs because they were included in the Amplify study. 16 Both patients stopped anticoagulant treatment after 6 months.

### Bleeding

**Fig. 2:**
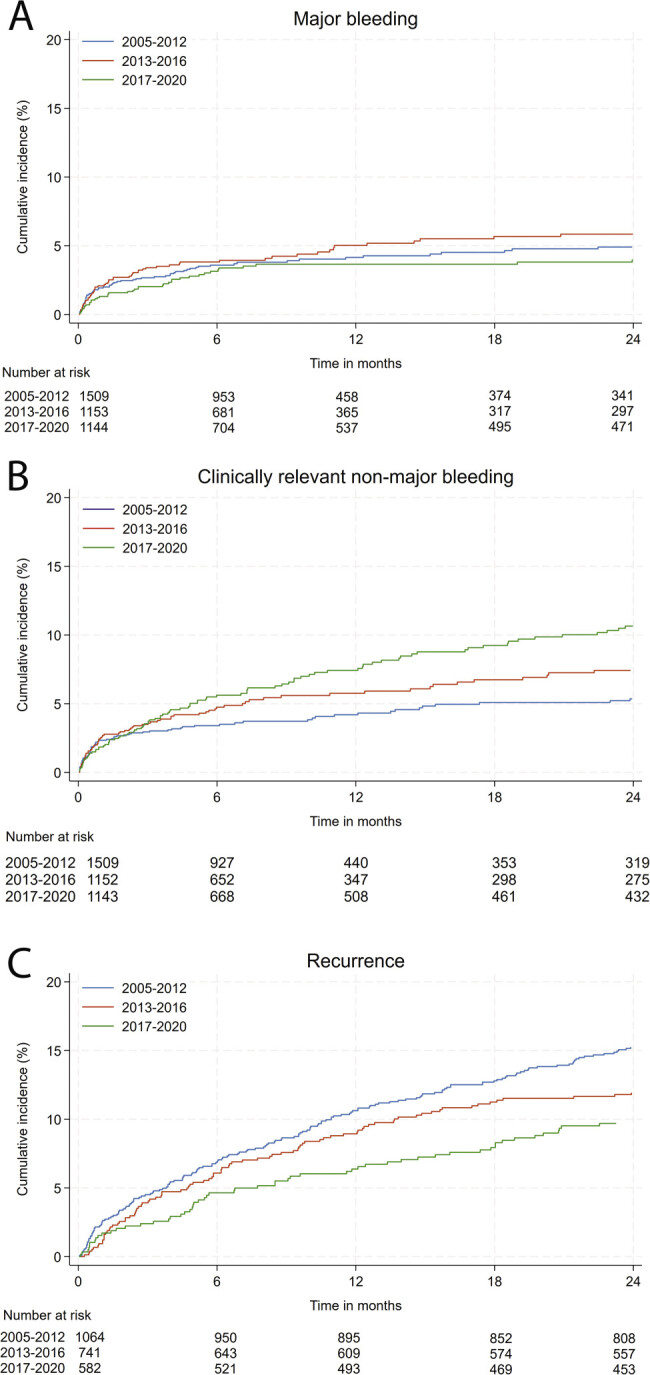
Cumulative incidence graphs of major bleeding ( **A** ) and clinically relevant nonmajor bleeding ( **B** ) on treatment, as well as recurrent VTE after treatment cessation ( **C** ).

A total of 158 (4.1%) of 3,812 patients who were treated with anticoagulants suffered MB (including 19 fatal, 0.5% of all patients, case-fatality 12%) during a follow-up of 3,518.4 patient-years. The MB rate for the whole study period was 4.5 (95% CI: 3.8–5.2) per 100 patient-years ( Fig. 2A ; Table 2 ). The lowest MB rate was observed during period 3 (3.1 per 100 patient-years), whereas the highest MB rate was observed during period 2 (5.6 per 100 patient-years; Table 2 ). Most MB ( *n* = 128) occurred within the first months of treatment, with a 6-month cumulative incidence of 3.5% (95% CI: 3.0–4.2). The most frequent MB sites were gastrointestinal, intracerebral, and genitourinary ( Table 3 ). Intracerebral bleedings accounted for around 20% of MBs during all three time periods. Fatal bleedings accounted for 21% of MBs during period 1 and 10% of MBs during period 3.

**Table 2:** Major and clinically relevant nonmajor bleeding events, event rates, and cumulative incidence for patients on treatment

	All patients, *n* = 3,812		2005–2012, *n* = 1,510		2013–2016, *n* = 1,154		2017–2020, *n* = 1,148	
	Events		Events		Events		Events	
Major bleeding								
Incidence rate per 100/person-y (95% CI)	158	4.5 (3.8–5.2)	63	5.0 (3.9–6.4)	56	5.6 (4.3–7.3)	39	3.1 (2.3–4.2)
6-m cumulative incidence, % (95% CI)	128	3.5 (3.0–4.2)	52	3.6 (2.7–4.6)	43	3.8 (2.8–5.1)	33	3.1 (2.2–4.3)
1-y cumulative incidence, % (95% CI)	17	4.3 (3.6–5.0)	5	4.2 (3.2–5.3)	8	5.0 (3.8–6.5)	4	3.7 (2.6–5.0)
2-y cumulative incidence, % (95% CI)	13	4.9 (4.2–5.7)	6	4.9 (3.8–6.2)	5	5.9 (4.4–7.5)	2	4.0 (2.9–5.4)
Clinically relevant nonmajor bleeding								
Incidence rate per 100/person-y (95% CI)	228	6.8 (5.9–7.7)	66	5.4 (4.3–6.9)	69	7.3 (5.7–9.2)	93	7.7 (6.3–9.5)
6-m cumulative incidence, % (95% CI)	159	4.4 (3.8–5.1)	60	3.4 (2.6–4.4)	51	4.8 (3.6–6.1)	58	5.6 (4.3–7.1)
1-y cumulative incidence, % (95% CI)	29	5.6 (4.9–6.5)	7	4.2 (3.2–5.4)	8	5.8 (4.4–7.3)	14	7.4 (5.9–9.2)
2-y cumulative incidence, % (95% CI)	40	7.6 (6.7–8.6)	9	5.4 (4.2–6.8)	10	7.4 (5.8–9.3)	21	10.7 (8.7–12.9)

Note: Cumulative incidences were calculated with death as a competing risk.

**Table 3:** Number of major, fatal, and clinically relevant nonmajor bleeding (CRNMB). Bleeding locations grouped into major and CRNMB events, with numbers and percentages

	All treated patients	2005–2012	2013–2016	2017–2020
Total number	3,812 a	1,510	1,154	1,148
Major bleeding				
Total, *n* (%)	158 (4.1)	63 (4.2)	56 (4.9)	39 (3.4)
Intracerebral, *n* (%)	31 (0.8)	12 (0.8)	11 (1.0)	8 (0.6)
Gastrointestinal, *n* (%)	48 (1.2)	16 (1.1)	18 (1.6)	14 (1.2)
Genitourinary, *n* (%)	13 (0.3)	7 (0.5)	4 (0.3)	2 (0.2)
Airways, *n* (%)	6 (0.1)	2 (0.1)	2 (0.2)	2 (0.2)
Other, *n* (%)	60 (1.6)	26 (1.7)	21 (1.8)	13 (1.1)
Fatal bleeding, c *n* (%)	19 (0.5)	7 (0.5)	8 (0.7)	4 (0.3)
Clinically relevant nonmajor bleeding				
Total, *n* (%)	228 (6.0)	66 (4.4)	69 (6.0)	93 (8.1)
Gastrointestinal, *n* (%)	36 (0.9)	12 (0.8)	12 (1.0)	12 (1.0)
Genitourinary, *n* (%)	81 (2.1)	18 (1.2)	23 (2.0)	40 (3.5)
Airways, *n* (%) c	31 (0.8)	9 (0.6)	9 (0.8)	13 (1.1)
Other, *n* (%)	80 (2.1)	27 (1.8)	25 (2.2)	28 (2.4)

a No treatment: 15, Thrombolysis or unfractionated heparin only: 10, unknown: 2.

b Refers to fatal bleeding.

c Only refers to airways.

A total of 228 of 3,812 patients had CRNMB, with a bleeding rate of 6.8 (95% CI: 5.9–7.7) per 100 patient-years ( Table 2 ). The CRNMB rate increased from 5.4 (95% CI: 4.3–6.9) per 100 patient-years during period 1 to 7.7 (95% CI: 6.3–9.5) per 100 patient-years for those who had a VTE during period 3, sHR: 1.87 (95% CI: 1.36–2.56); *p* < 0.001 for a difference between groups ( Fig. 2B ; Table 4 ). The 6-month cumulative incidence of CRNMB was 3.4% (95% CI: 2.6–4.4) for patients who had a VTE during period 1 and 5.6% (95% CI: 4.3–7.1) for patients who had a VTE during period 3, while the 2-year cumulative incidence increased from 5.4% (95% CI: 4.2–6.8) to 10.7% (95% CI: 8.7–12.9). Genitourinary bleeding was the most common CRNMB, affecting 2.1% of all patients, increasing from 1.2 to 3.5% over time ( Table 3 ).

**Table 4:** Subhazard ratios for the risk of major bleeding, clinically relevant nonmajor bleeding, and recurrence during different time periods, with 2005 to 2012 as reference

	Subhazard ratios (95% confidence interval)	*p* -Value
Major bleeding		
2005–2012	1 (reference)	
2013–2016	1.18 (0.82–1.68)	0.38
2017–2020	0.82 (0.55–1.22)	0.32
Clinically relevant nonmajor bleeding		
2005–2012	1 (reference)	
2013–2016	1.38 (0.98–1.94)	0.06
2017–2020	1.87 (1.36–2.56)	<0.001
Recurrence after treatment cessation		
2005–2012	1 (reference)	
2013–2016	0.69 (0.58–0.83)	<0.001
2017–2020	0.51 (0.41–0.65)	<0.001

### Recurrent VTE

A total of 669 (28.0%) of 2,387 patients who discontinued anticoagulant treatment had a recurrent VTE (including 11 fatal cases, case fatality 1.6%) during a follow-up of 13,985.5 patient-years ( Table 5 ). The recurrence rate after treatment discontinuation was 5.1 (95% CI: 4.6–5.6) per 100 patient-years for patients who had a VTE during period 1 and 3.9 per 100 patient-years (95% CI: 3.2–4.9) for patients who had a VTE during period 3, sHR: 0.51 (95% CI 0.41–0.65); *p* < 0.001 for a difference between groups ( Fig. 2C ; Table 4 ). The 2-year cumulative recurrence was 15.3% (95% CI: 13.2–17.5) for patients with a VTE during period 1 and 9.7% (95% CI: 7.5–12.3) during period 3. The most common type of recurrence was DVT (319 events), followed by PE (317 events; Supplementary Table S1 ). Recurrence during treatment was reported in 113 of 3,812 patients (3%; Supplementary Table S2 ), with a decreasing trend from 4% of patients with a VTE in period 1 to 2% in period 3. Four of 113 recurrences during anticoagulant treatment were fatal events (case-fatality 3.5%).

**Table 5:** Recurrent venous thromboembolism (VTE) events following treatment cessation for all patients were grouped based on the VTE diagnosis date

	All patients		2005–2012		2013–2016		2017–2020	
	Events		Events		Events		Events	
Incidence rate per 100/person-y (95% CI)	669	4.8 (4.4–5.2)	406	5.1 (4.6–5.6)	179	4.6 (3.9–5.3)	84	3.9 (3.2–4.9)
6-mo cumulative incidence, % (95% CI) a	145	6.1 (5.2–7.1)	73	6.9 (5.5–8.5)	45	6.1 (4.5–8.0)	27	4.7 (3.1–6.6)
1-y cumulative incidence, % (95% CI) a	71	9.1 (8.0–10.3)	40	10.6 (8.9–12.6)	21	8.9 (7.0–11.1)	10	6.4 (4.6–8.6)
2-y cumulative incidence, % (95% CI) a	90	12.9 (11.6–14.3)	49	15.3 (13.2–17.5)	22	11.9 (9.7–14.4)	19	9.7 (7.5–12.3)

a Events, event rates, and cumulative incidence. Cumulative incidences calculated with death as a competing risk.

## Discussion

In this single-center registry study evaluating bleeding and recurrence patterns among cancer-free patients with VTE from 2005 to 2020, the rate of MB during anticoagulant treatment demonstrated no clear temporal trend, whereas the incidence of CRNMB increased over time, paralleling increasing use of DOACs. The recurrence rate after discontinuation of anticoagulant treatment decreased as the use of indefinite treatment increased, likely leaving fewer patients with high recurrence risk untreated.

While the rate of MB was largely stable across the time periods when compared with period 1, a notable reduction was observed in period 3. This finding could be explained by an increased use of DOACs, which have been shown to be associated with reduced MB in both phase 3 studies 16 and register data. 17 18 However, the highest MB rates in the present study were observed in patients with VTE in period 2, when 53% of patients were prescribed DOACs, which is not in line with this explanation. Another explanation for the low MB rates in period 3 might be the potential increase in the prescription of reduced-dose DOACs. However, this remains speculative due to the lack of dosage data in the registry. Our results on unchanged MB rates align with a previous study comparing the two COMMAND-VTE registers: 2010 to 2014 (register 1, warfarin dominance) and 2015 to 2020 (register 2, DOAC dominance). 7 In contrast, another study reported a decrease in bleeding events between 2011 and 2020, as treatment patterns changed. 8 Key differences include the use of the ISTH-BAT system for bleeding assessment in that study, with a cutoff of 3 points. The ISTH-BAT system was not designed to assess bleeding in patients on anticoagulant therapy, and the lack of subdivided bleeding points means that the included bleedings may vary widely in severity, thereby limiting the validity of temporal comparisons. 19 In contrast, the standardized ISTH bleeding definition was used in the present study. 13 14

We observed an increase over time in CRNMB, which was unexpected given that DOACs were associated with lower CRNMB event rates than warfarin in clinical trials, 20 and with rates comparable to warfarin in a GARFIELD-VTE register study. 21 However, the single most common bleeding type among CRNMB events in the present study was genitourinary bleeding, accounting for most of the increase over time. Factor Xa inhibitors, in particular rivaroxaban, which was the most frequently used DOAC in the present study, have been shown to increase the risk of uterine bleeding and hematuria compared with VKAs. 22 23 24 The increase in CRNMB may also be due to increased use of indefinite treatment across a broader patient population, leading to more bleeding-prone patients receiving longer treatment durations. Given the lack of data on several comorbidities and renal function, we could not account for differences in comorbidities across time periods.

The observed decline in recurrence rate after treatment discontinuation likely reflects the increasing use of indefinite treatment for patients at high risk of recurrence, thereby selecting patients with lower recurrence risk who subsequently discontinue treatment. Our results on declining recurrence rates align with previous studies. 7 8

The main question is whether changes in treatment practice are beneficial to patients, which, given the results of this study, could essentially be determined by the impact of recurrence versus CRNMB. International guidelines recommend indefinite anticoagulation for patients with unprovoked VTE without a high bleeding risk. 1 2 25 However, the benefit of this is uncertain both in terms of mortality, quality-adjusted life years, and cost-effectiveness. 26 Additionally, the optimal ratio between bleeding risk and the reduction in recurrence risk remains uncertain. 27

### Strengths and Limitations

The main strength of this study is the large number of unselected VTE patients with validated initial and recurrent VTE events, as well as validated bleeding events, all classified according to the ISTH standard definitions. Recurrent VTE is challenging to study in national health registries due to low coding validity for recurrent events. 11 In this context, our study provides more reliable real-world evidence. Furthermore, by accounting for death as a competing risk in the bleeding and recurrence analysis, our study yields more precise and clinically relevant estimates.

The study also has several limitations. First, this is a single-center study in a high-income country with low-cost public health care, making generalizability to patients in other countries or health care systems uncertain. Additionally, we lack data on DOAC dosage, which has been shown to influence bleeding rates, while recurrence rates on treatment are low regardless of dosage. 28 Patients who experienced a bleeding or recurrent event outside the Østfold region would not have been registered for the acute event in our register; however, such an event is likely to be recorded in the hospital's records during follow-up or at a later visit. Fatal events due to bleeding or VTE recurrence abroad or in another Norwegian region are registered only as deaths, since our register does not include data from the National Cause of Death register. However, the date of death is automatically obtained from the National Population Registry. We do not have data on bleeding after treatment discontinuation, which would have strengthened our analysis of risk versus benefit with the change in treatment guidelines.

## Conclusion

Over the past decade, changes in anticoagulant management have not led to a clear temporal shift in MB rates but have resulted in an increase in CRNMB. Recurrences after treatment discontinuation have declined, likely due to the selection of low-risk patients for discontinuation, while those at higher risk of recurrence continue on indefinite treatment.

## Notes

**What Is Known on This Topic?**

- Vitamin K antagonists have been replaced by direct oral anticoagulants (DOACs) for the treatment of VTE, with indefinite treatment now recommended more liberally.
- The effects on MB and clinically relevant non-MB during anticoagulant treatment, and on recurrences after anticoagulant discontinuation, remain inadequately studied.

**What Does This Paper Add?**

- With increasing use of DOACs and indefinite treatment, MB rates did not exhibit a clear temporal trend, while clinically relevant non-MB rates increased.
- Recurrence rates after treatment discontinuation decreased, likely due to the more liberal use of indefinite treatment in patients at high risk for recurrence.
