# Supplementary material for: Bleeding and Recurrence in Patients with Venous Thromboembolism with Changing Anticoagulant Treatment Patterns: Findings from the TROLL Registry
Source: TH Open. 2026 Mar 24;10:a28328297. doi: 10.1055/a-2832-8297 (PMC13062681; doi:10.1055/a-2832-8297)
Supplement: Supplementary file 1 — Supplementary Material [file 10-1055-a-2832-8297_28407890.pdf]

Supplementary Table 1. Type of recurrence after treatment cessation. Fatal recurrence was defined as death within one week of recurrence.

**Type of recurrence**	**All patients**	**2005-2012**	**2013-2016**	**2017-2020**
PE	317	184	91	42
DVT	319	200	81	38
UEDVT	17	11	4	2
Abdominal or cerebral	16	11	3	2
Fatal recurrence	11	9	2	0

Abbreviations: PE = pulmonary embolism, DVT = deep vein thrombosis, UEDVT = upper-extremity deep vein thrombosis.

Supplementary Table 2. Recurrence during anticoagulant treatment, number, and percentage of all treated patients.

	**All patients**	**2005-2012**	**2013-2016**	**2017-2020**
Recurrence during AC, n(%)	113 (3)	62 (4)	25 (2)	26 (2)
Fatal recurrence during AC (n)	4	3	1	0

Abbreviation: AC = anticoagulant treatment.

TH Open | DOI 10.1055/a-2832-8297 | © The Author(s).
